# On the Relationship between Sialomucin and Sulfomucin Expression and Hydrogenotrophic Microbes in the Human Colonic Mucosa

**DOI:** 10.1371/journal.pone.0024447

**Published:** 2011-09-09

**Authors:** Jennifer A. Croix, Franck Carbonero, Gerardo M. Nava, Mark Russell, Eugene Greenberg, H. Rex Gaskins

**Affiliations:** 1 Division of Nutritional Sciences, University of Illinois at Urbana-Champaign, Urbana, Illinois, United States of America; 2 Department of Animal Sciences, University of Illinois at Urbana-Champaign, Urbana, Illinois, United States of America; 3 Department of Internal Medicine, University of Illinois at Urbana-Champaign, Urbana, Illinois, United States of America; 4 Department of Pathobiology, University of Illinois at Urbana-Champaign, Urbana, Illinois, United States of America; 5 Institute for Genomic Biology, University of Illinois at Urbana-Champaign, Urbana, Illinois, United States of America; 6 Carle Foundation Hospital Institute for Digestive Health, Urbana, Illinois, United States of America; Charité-University Medicine Berlin, Germany

## Abstract

The colonic mucus layer is comprised primarily of acidomucins, which provide viscous properties and can be broadly classified into sialomucins or sulfomucins based on the presence of terminating sialic acid or sulfate groups. Differences in acidomucin chemotypes have been observed in diseases such as colorectal cancer and inflammatory bowel disease, and variation in sialo- and sulfomucin content may influence microbial colonization. For example, sulfate derived from sulfomucin degradation may promote the colonization of sulfate-reducing bacteria (SRB), which through sulfate respiration generate the genotoxic gas hydrogen sulfide. Here, paired biopsies from right colon, left colon, and rectum of 20 subjects undergoing routine screening colonoscopies were collected to enable parallel histochemical and microbiological studies. Goblet cell sialo- and sulfomucins in each biopsy were distinguished histochemically and quantified. Quantitative PCR and multivariate analyses were used to examine the abundance of hydrogenotrophic microbial groups and SRB genera relative to acidomucin profiles. Regional variation was observed in sialomucins and sulfomucins with the greatest abundance of each found in the rectum. Mucin composition did not appear to influence the abundance of SRB or other hydrogenotrophic microbiota but correlated with the composition of different SRB genera. A higher sulfomucin proportion correlated with higher quantities of *Desulfobacter*, *Desulfobulbus* and *Desulfotomaculum*, relative to the predominant *Desulfovibrio* genus. Thus, acidomucin composition may influence bacterial sulfate respiration in the human colon, which may in turn impact mucosal homeostasis. These results stress the need to consider mucus characteristics in the context of studies of the microbiome that target intestinal diseases.

## Introduction

The colonic mucus layer is an important interface between the host epithelium and the luminal contents, including the mutualistic microbiota. Mucins, which are largely responsible for the viscous properties of the mucus layer, consist of a peptide backbone containing alternating glycosylated and non-glycosylated domains [1]. Mucins can be broadly classified into neutral and acidic chemotypes, which are categorized further into sialomucins or sulfomucins based on the presence of terminal sialic acid or sulfate groups on the oligosaccharide chain, respectively [2]. Previous studies using high iron diamine and alcian blue (HID/AB) staining reported an overall predominance of sulfomucin in the human colon with distinct patterns of sialomucin and sulfomucin distribution both regionally and along the crypt [2], [3]. However, quantitative measurements of sialomucin and sulfomucin have not been reported, and there is little information on the extent of interindividual variation in acidomucin chemotypes in healthy individuals.

Mucin oligosaccharide chains are structurally heterogeneous [4], [5]. This heterogeneity influences the biological and physiological properties of mucins as well as the types of bacteria able to colonize the mucus layer [6]–[8], as carbohydrates and terminal groups on mucin oligosaccharide chains can serve both as binding sites and as a source of nutrients for microbes. Particular focus is given here to sulfate-reducing bacteria (SRB), members of the normal hydrogenotrophic colonic microbiota, which generate the gas hydrogen sulfide (H_2_S) via sulfate respiration [9]. There is considerable evidence implicating SRB and H_2_S in the pathogenesis of chronic inflammatory disorders of the colon [10], [11], including the observation of increased numbers of SRB in stool of ulcerative colitis (UC) patients compared to controls and in UC patients with active disease compared to those in remission [12]. In addition, H_2_S is a potent genotoxin [13] and thus may be linked to sporadic colorectal cancer. Relatively little is known about the ecology of colonic SRB populations in the human colon; however, in the mouse, SRB populations were found to be most abundant in those intestinal regions harboring the greatest density of sulfomucin-containing goblet cells [14]. Furthermore, studies in mice suggest that the sulfate utilized by sulfate reducers may be derived from sulfomucins rather than exogenous sulfate [15]. Thus, it can be hypothesized that acidomucin chemotype composition and SRB population abundance are interrelated.

The aim of the present study was to better understand spatial and interindividual variation in sialomucins and sulfomucins and the relationship between acidomucin chemotype composition and SRB in the human colon. Sialomucins and sulfomucins were quantified and scored in biopsies from right colon, left colon, and rectum. Real-time quantitative PCR (qPCR) targeting functional genes was used to quantify the SRB population and the two other potentially competing hydrogenotrophic groups, acetogens and methanogenic Archaea (MA), as well as four different SRB genera targeting 16S rRNA genes in a matched set of biopsies. The combined data were subjected to multivariate analyses to elucidate possible relationships between acidomucin chemotypes and hydrogenotrophic microbiota.

## Results

### Mucin histochemistry

**Figure 1:**
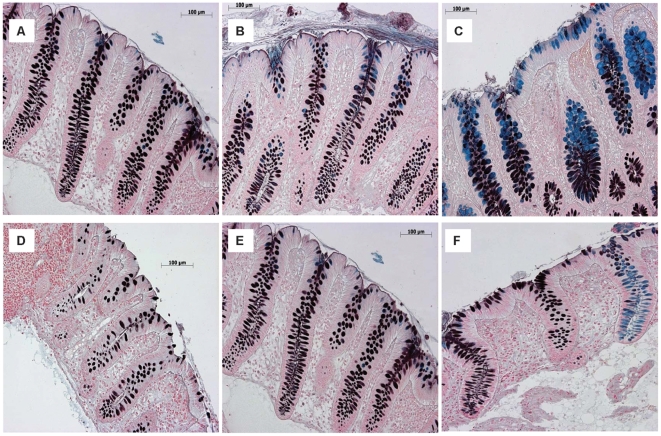
High iron diamine and alcian blue (pH 2.5) stained sections of human colon. Sulfomucin is stained black/brown and sialomucin is stained blue. Top 3 panels demonstrate typical spatial variation among 3 colonic regions within one individual: **A**) Right colon **B**) Left colon **C**) Rectum. Bottom 3 panels demonstrate typical interindividual variation within each colonic region. In this case, all 3 are sections of right colon from **D**) Subject 6 **E**) Subject 8 **F**) Subject 9.

Both spatial and interindividual variation in sialomucin and sulfomucin content were evident in the HID/AB-stained biopsy sections (**Figure 1**). In general, goblet cells in the right colon were predominantly sulfomucin-positive with a few sialomucin-positive goblet cells near the surface epithelium and occasionally in the lower crypt. Goblet cell mucins in the left colon were predominantly sulfated with variable numbers of sialomucin-positive goblet cells found in the upper crypt and surface epithelium. In the rectum, an increase in the density of sialomucin in the surface epithelium and upper crypt was found consistently among individuals. The lower crypt in the rectum contained primarily sulfomucin-positive goblet cells. Interindividual variation included differences in the abundance of sulfomucin and sialomucin and in their spatial distribution across regions of the lower intestine and along the crypt axis.

### Sialomucin and sulfomucin abundance

**Figure 2:**
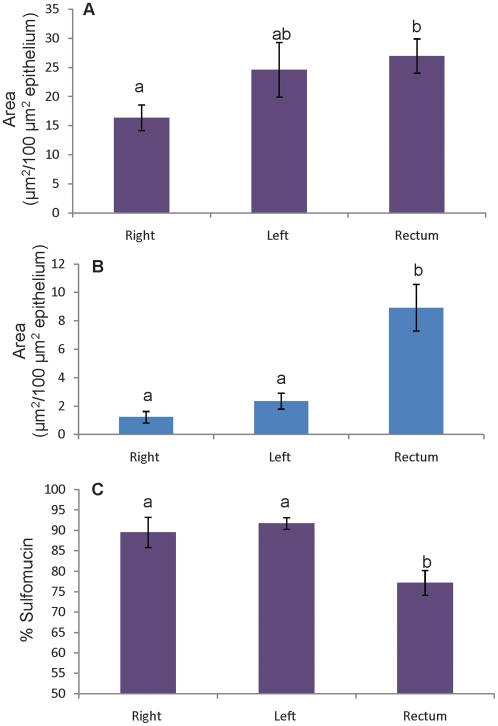
Area of acidomucin staining per area of epithelium in biopsies from three regions of colon. **A**) Sulfomucin and **B**) sialomucin staining per area of epithelium and **C**) % sulfomucin in each colonic region. All data represent mean ± SEM (n = 20); statistically significant differences are indicated by different letters (p<0.05).

To better understand regional and interindividual differences in sialo- and sulfomucin abundance, the area of sialo- and sulfomucin staining within goblet cells was measured per area of epithelium analyzed. Data obtained from quantitative analysis of sialomucin and sulfomucin staining in all 60 biopsies as well as the percent of stained mucin that stained as sulfomucin (% sulfomucin = sulfomucin area/[sulfomucin area + sialomucin area]) revealed that sulfomucin-containing goblet cells were present in all biopsies collected and that sulfomucin abundance was greater than sialomucin abundance in all biopsies with the exception of right colon from one subject and rectum from another. Comparison of sialomucin and sulfomucin among the three colonic regions for all subjects revealed both mucin chemotypes to be most abundant in the rectum but not significantly different between right and left colon and that, for sulfomucin abundance, left colon and rectum did not differ significantly (**Figure 2A and B**). Analysis of % sulfomucin revealed sulfomucins to be predominant in all three regions (**Figure 2C**) but less so in the rectum, where the presence of sialomucins was increased.

**Figure 3:**
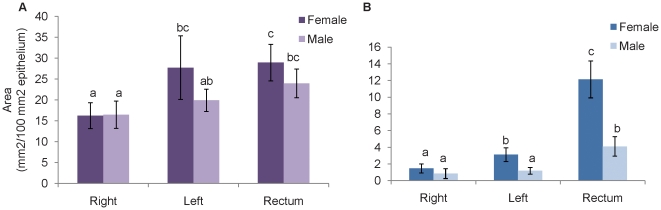
Comparison between female (n = 12) and male (n = 8) subjects of sulfomucin to sialomucin staining. Area of **A**) sulfomucin and **B**) sialomucin staining in right colon, left colon, and rectum biopsies. The data represent mean ± SEM; statistically significant differences are indicated by different letters (p<0.05).

A comparison of sialomucin and sulfomucin abundance between male and female subjects revealed that, in general, both acidomucins increased in abundance from proximal to more distal colonic regions in female subjects and to a lesser extent in males (**Figure 3**). Sulfomucin was similar between men and women in all three regions (**Figure 3A**). Male subjects, while demonstrating significantly higher sialomucin abundance in rectum than right or left colon, had a similar level in rectum to left colon in females. Although the abundance of the two acidomucin chemotypes was similar between male and female subjects for other colonic regions, female subjects harbored significantly more sialomucin in the rectum than male subjects (**Figure 3B**).

### Sulfomucin and sialomucin distribution along the crypt

**Figure 4:**
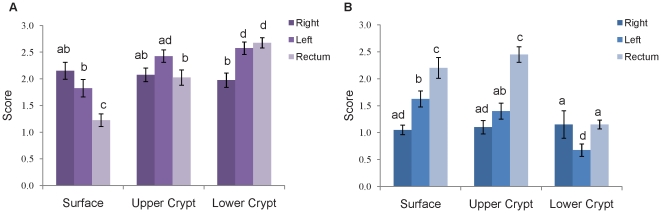
Acidomucin scores for the surface epithelium, upper crypt, and lower crypt of the right colon, left colon, and rectum. **A**) Sulfomucin and **B**) sialomucin scores were assigned based on the percent positive staining for each acidomucin as follows: 0 = no mucin staining in epithelium; 1 = 1–10% mucin staining; 2 = 11–50%; 3 = >50%. The data represent mean ± SEM (n = 20); statistically significant differences are indicated by different letters (p<0.05).

To define sulfomucin and sialomucin gradients along the crypt semi-quantitatively, sulfo- and sialomucin staining were scored along the surface epithelium, upper crypt, and lower crypt. Score comparisons for each region are shown in **Figure 4**. For sulfomucin, scores were highest in the lower crypt of the left colon and rectum and lowest for the surface epithelium of the rectum. Thus, the greater abundance of sulfomucin in the rectum likely reflects more sulfomucin in the lower crypt. A trend towards decreasing sulfomucin on the surface epithelium in more distal regions of the colon was also observed. Sialomucin scores were highest in the surface epithelium and upper crypt of the rectum, indicating that the greater overall area of rectal sialomucin reflects increasing amounts in those regions of the crypt. Both surface and upper-crypt sialomucin appeared to increase for more distal sections. Sialomucin scores were lowest in the lower crypt of the left colon.

### Relationship between mucin chemotypes and microbiota

**Figure 5:**
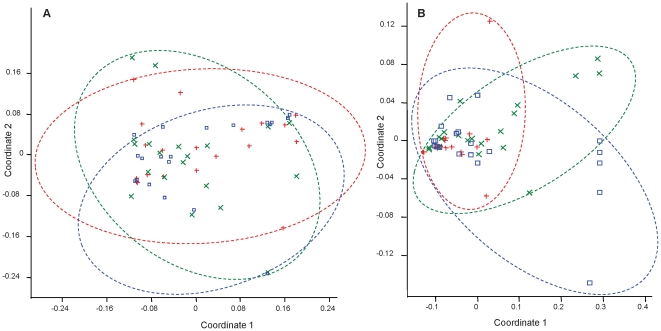
Non-metric multidimensional scaling of acidomucin abundance relative to abundance of hydrogenotrophic microbes. **A**) Abundance of sulfomucin versus that of acetogens, methanogenic Archaea and sulfate-reducing bacteria and **B**) Abundance of sulfomucin versus that of *Desulfobacter*, *Desulfobulbus*, *Desulfotomaculum* and *Desulfovibrio*. Right colon samples appear in red, left colon in green, and rectum in blue.

Functional genes for the three hydrogenotrophic groups; acetogens, methanogenic Archaea (MA) and SRB; were persistently detected in the 90 biopsies. Clustering by subject or region was not observed, but on average MA relative abundance increased and SRB decreased in a proximal to distal gradient. Significant clustering by region was not observed when combining absolute or relative mucin chemotype abundance with functional gene abundance as shown by NMDS (**Figure 5A**). Multivariate ANOVA confirmed this trend. Contrastingly, significant regional clustering was observed with the abundance of different SRB genera (**Figure 5B**). Multivariate ANOVA further indicated significant differences between rectum and right colon for *Desulfovibrio* combined with sulfomucin area (p<0.05) and between left colon and rectum as well as right colon for *Desulfobacter* combined with sulfomucin area (p<0.05).

**Figure 6:**
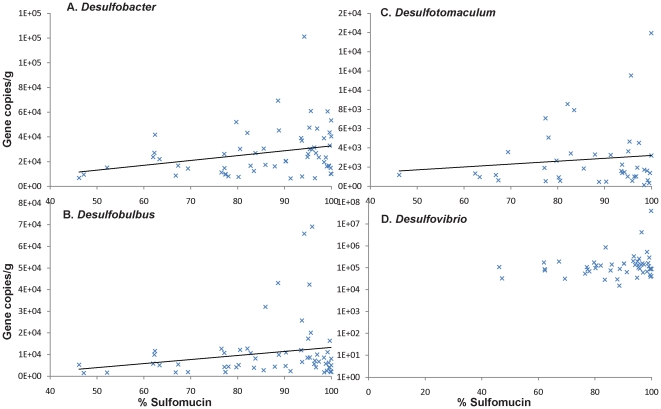
Abundance of four genera of SRB compared to relative abundance of sulfomucins (% of mucin chemotype). **A**) *Desulfobacter* **B**) *Desulfobulbus* **C**) *Desulfotomaculum* and **D**) *Desulfovibrio* (gene copies per g). Linear regression curve is shown for A), B) and C).

Significant correlations (p<0.05) were further detected between the relative percentage of sulfomucin and *Desulfobacter* abundance. *Desulfobulbus* abundance also correlated with *Desulfobacter* abundance. The abundance of the four SRB genera was plotted against the sulfomucin relative abundance; *Desulfobacter*, *Desulfobulbus* and to a lesser extent *Desulfotomaculum* all increased in abundance with increasing relative sulfomucin abundance (**Figure 6**). In contrast, this relationship was not observed for *Desulfovibrio*, the predominant SRB genus.

Multivariate analysis was used to examine data on the diversity of SRB using a molecular fingerprinting approach [16] together with the various mucin parameters. Differing SRB genotypes were found to be associated with specific individuals, and those clusters were not altered by inclusion of mucin data (data not shown).

## Discussion

In the present study a quantitative analysis of goblet cell sialomucins and sulfomucins in human colon biopsies was performed and compared with an analysis of microbiota abundance from matched biopsies collected from the same colonic regions in each subject. Sialomucin and sulfomucin were most abundant in the rectum, and sulfomucin was the predominant mucin chemotype in all three regions except for two biopsies. The increase in sialomucins in rectum compared to right colon and an overall predominance of sulfomucin in the human colon is consistent with previously described observations [2]. Sulfomucin may offer additional protection from luminal insults by increasing mucus viscosity as well as resistance to bacterial degradation and microbe adhesion [7]. Thus, individuals who have either a lower absolute abundance or lower predominance of sulfomucin relative to sialomucin may be at greater risk from the effects of luminal substances with the potential to cause disease.

Intriguingly, a significant increase was observed for sialomucin abundance in the rectum of female subjects. Sulfomucin abundance, on the other hand, was similar in the three colonic regions between male and female subjects. The greater abundance of sialomucin in the female rectum could possibly reflect hormonal regulation of rectal sialomucin production, as colonic sialomucin was previously found to increase during the late progesterone phase of the menstrual cycle [17]. Despite the fact that the average age of female subjects in this study was 55.4 years, compared to the average age of 51 years for menopause in U.S. women, a difference in circulating hormone levels between genders might account for this effect. The observed gender difference in rectal sialomucin levels would require additional subject data as well as a larger cohort of subjects to determine the consistency of this observation and its possible cause.

Scoring of the abundance of each mucin chemotype along the surface epithelium confirmed previous qualitative descriptions of mucin chemotype distribution along the crypt, including a greater number of sialomucin-containing goblet cells in the upper crypt of the rectum [2]. The increase in sulfomucin abundance measured in the rectum compared to right colon was due mainly to increased sulfomucin in the lower crypt rather than in the upper crypt or surface epithelium. This fine-scale mapping is noteworthy as mucins in surface and upper crypt goblet cells may be more important in mediating host microbe interactions, while disease-related changes in mucin chemotypes may initially be observed in the lower crypt with less differentiated goblet cells.

Alterations in sialo- and sulfomucin chemotypes have been observed in patients with inflammatory bowel disease and colorectal cancer [18]. Specifically, a significant decrease in sulfomucins and an increase in sialomucins have been observed in UC and were found to correlate with disease severity [8], [19]–[23]. In the case of colorectal cancer, a similar change is seen in transitional mucosa and non-mucinous adenocarcinomas [24]. The present quantitative data for sialomucin and sulfomucin abundance provides a framework for the study of acidomucin chemotypes in disease by defining the normal range for each chemotype.

While quantitative analysis of sialo- and sulfomucin abundance and distribution along the crypt revealed spatial and interindividual variation in these two mucin chemotypes, the factors that contribute to this variation remain to be defined. It is likely that both environmental and genetic components are involved. Previously reported heterogeneity in *O*-acetylation of sialomucin is believed to reflect genetic differences in expression of an *O*-acetyltransferase [18]. Thus, interindividual differences in alleles encoding specific sialo- and/or sulfotransferases may be similarly involved in determination of sulfo- and sialomucin expression.

Potential links between colonic acidomucin chemotypes and commensal microbiota are, to our knowledge, assessed for the first time in this study. In contrast with our initial hypothesis, neither absolute nor relative abundance of sulfomucin was correlated with SRB abundance. Although sulfomucins represent a potentially important source of sulfate, various other sources exist in the human colon. In addition, SRB can survive without sulfate respiration in natural environments [24], and the possibility of acetogenic lifestyles for SRB has not been studied in intestinal ecosystems. However, a significant shift in SRB genera might be driven by sulfomucin abundance, with the three less abundant genera decreasing with decreasing sulfomucin abundance. This may indicate that *Desulfovibrio*, the major SRB genus as observed in this study and others [25]–[27], are able to thrive without sulfomucin-derived sulfate. Contrastingly, *Desulfobacter*, *Desulfobulbus* and *Desulfotomaculum* may be more dependent on sulfate derived from sulfomucins. Differences in access to exogenous sulfate or syntrophic associations with mucin-degrading bacteria may explain this trend. All these SRB genera include species capable of sulfate respiration combined with dihydrogen and carbon dioxide consumption, which is the major metabolism postulated for SRB in the colon. However, hydrogenotrophy appears to be a marginal pathway for environmental isolates of *Desulfobacter* and *Desulfobulbus*, and some *Desulfobulbus* have been shown to be facultative sulfate reducers [28], [29]. *Desulfovibrio piger*, the most often detected SRB in the human colon, is a strict hydrogenotroph, but other *Desulfovibrio* species are facultative hydrogenotrophs [30]. Studies on colonic SRB metabolism will be needed to better understand the importance of sulfomucin in the mucosal niche.

In summary, this study provides the first quantitative assessment of regional and interindividual variation in sialo- and sulfomucins in healthy human subjects. In addition, it provides the first description of the relationship between SRB and specific acidomucin chemotypes. The shift in SRB genera driven by acidomucin proportion may result in altered metabolic pathways ultimately leading to chronic colonic inflammation or disease.

## Methods

### Sample Collection

All procedures were approved by the Carle Foundation Hospital and University of Illinois at Urbana-Champaign Institutional Review Boards. Twenty healthy subjects (12 women and 8 men) aged 47–64 years undergoing routine screening colonoscopy at Carle Foundation Hospital (Urbana, IL USA) were recruited for this study. Enrolled subjects had no known history of gastrointestinal disease and had not been on antibiotics for at least thirty days prior to sample collection. After obtaining written informed consent, mucosal biopsies were collected during a routine screening colonoscopy from the right colon, left colon, and rectum for mucin histochemistry, histopathological analysis, and microbial analysis. Right colon biopsies were taken approximately 5 cm distal to the ileocaecal valve; left colon was identified as the relatively straight area between splenic flexure and sigmoid colon, and biopsies were taken approximately midway; rectum biopsies were collected at approximately 18 cm distal to the anal opening as well as distal to the first rectal valve which is typically 5–15 cm from anal opening. Biopsies for microbial analysis were immediately frozen in liquid nitrogen and then stored until DNA extraction at −80°C. Biopsies for mucin histochemistry were fixed in Bouin's solution, while biopsies for histopathological examination were fixed in 10% neutral buffered formalin. Fixed biopsies were sent to the Carle Foundation Hospital Pathology Services Laboratory (Urbana, IL USA) for processing, embedding, and sectioning. Demographic information including age, gender, height, weight, race, and smoking were collected from review of subjects' medical charts. Subject demographic information and endoscopic findings are summarized in **Table 1**.

**Table 1:** Subject Characteristics and Endoscopic Findings.

Gender	12 Female, 8 Male
**Age (y)**	53.8 (1.1) [47–63.5]
**Height (cm)**	168.9 (1.84) [157–183]
**Body Weight (kg)**	83.7 (3.94) [55–118.2]
**BMI (kg/m^2^)**	27.5 (0.89) [21.3–33.2]
**Race (n)**	
African American	1
Caucasian	9
Unknown/Unspecified	10
**Smoking Behavior (n)**	
Non-smoker	16
Former smoker	2
Smoker	2
**Endoscopic Findings (n)**	
**Adenomatous Polyps**	3
**Diverticulosis**	3
**Hemorrhoids**	3
**Acute Inflammation**	2

Values are mean (SEM) [range] or number of subjects (n). Height and BMI data unavailable for subjects 8, 12, and 19.

### Mucin Histochemistry and Histopathological Analysis

To identify sialomucins and sulfomucins, biopsy sections were stained with high iron diamine (HID) and alcian blue (AB), pH 2.5, as previously described [14] and counterstained with nuclear fast red for 2 minutes. For histopathological analysis, sections were stained with hematoxylin and eosin and then examined by a board-certified pathologist.

### Quantification of Sialomucin and Sulfomucin

Images of HID/AB-stained sections were captured using a Zeiss Axiovert 200 M Microscope and the Mosaix module in Axiovision 4.5 software. The Automeasure module in Axiovision 4.5 was then used to select and quantify the areas of sialomucin and sulfomucin within goblet cells based on pixel color (shown for sulfomucin in **Figure S1**). For each biopsy, the area of sialomucin and sulfomucin within goblet cells was measured for the entire section with the exception of areas too damaged to distinguish goblet cells or epithelium. The area of each mucin chemotype was normalized to the area of epithelium containing the quantified mucin.

### Sialomucin and Sulfomucin Scoring

Positive staining for sialomucin and sulfomucin along the surface epithelium, upper crypt, and lower crypt was scored as follows: 0 = no mucin staining in epithelium; 1 = 1–10% mucin staining; 2 = 11–50%; 3 = >50%. Lower and upper crypt were designated as bottom half and upper half of visible crypt, respectively, while surface epithelium was considered those cells aligned perpendicular to crypt axis and not lining upper crypt collar. Scoring was performed at two independent time points for each image, and the two scores were averaged.

### DNA extraction and quantitative PCR analysis of hydrogenotrophic functional genes and SRB genera

Genomic DNA was extracted from biopsies using a commercial kit (QIAamp DNA Stool Mini Kit, Qiagen, Valencia, CA). Real-time qPCR was performed on all colonic DNA extracts (20 subjects) using the SYBR® Green PCR Master Mix (Applied Biosystems). Primers ACSF1/ACSR1 [31] and FTHFSf/FTHFSr [32] were used to target functional genes *acs* and *fhs* of acetogenic bacteria (phylogenetically diverse hydrogenotrophs). Primers ME1/ME2 [33] and DSR1fdeg/DSR4rdeg [34] were used for *mcrA* (MA) and *dsrA* (SRB) genes, respectively. Primer pairs targeting 16S rRNA genes of SRB genera *Desulfobacter*, *Desulfobulbus*, *Desulfotomaculum* and *Desulfovibrio* were used to quantify SRB genera [35]. Triplicates were run on a 7900HT Fast Real-Time PCR System (Applied Biosystems) using a dissociation curve. Standard curves were determined simultaneously using plasmids containing *dsrA*, *mcrA* and *fhs* genes or diluted PCR products from reference strains for *acs* and the 16S rRNA genes.

### Statistical analyses

Mucin chemotype abundance and qPCR results were concatenated to form a collective data set, and the resulting output files were used in multivariate statistical analyses using multivariate ANOVA (MANOVA) and non-metric multidimensional scaling analysis (NMDS). The results of the NMDS ordinations were corroborated by a non-parametric clustering technique, multivariate cluster analysis (MCA). All these statistical tests were carried out using the Morisita index for quantitative data. All multivariate statistical analyses were performed with the PAST software package [36].

## Supporting Information

Figure S1 **Image analysis of mucin area.** A) Images were captured at 200× magnification using a Zeiss Axiovert 200 M Microscope and Axiovision 4.5 software. The MosaiX module was used to scan the entire area of the section at 200× magnification and generate a single image. B) The Automeasure module was used to select and quantify an area of sulfomucin (outlined in green) based on parameters that were used to define sulfomucin staining. C) Selection by software (in green) of total sulfomucin area in the entire section.
(TIF)
