# Supplementary figures and images for: On the Relationship between Sialomucin and Sulfomucin Expression and Hydrogenotrophic Microbes in the Human Colonic Mucosa
Source: PLoS One. 2011 Sep 9;6(9):e24447. doi: 10.1371/journal.pone.0024447 (PMC3170330; doi:10.1371/journal.pone.0024447)

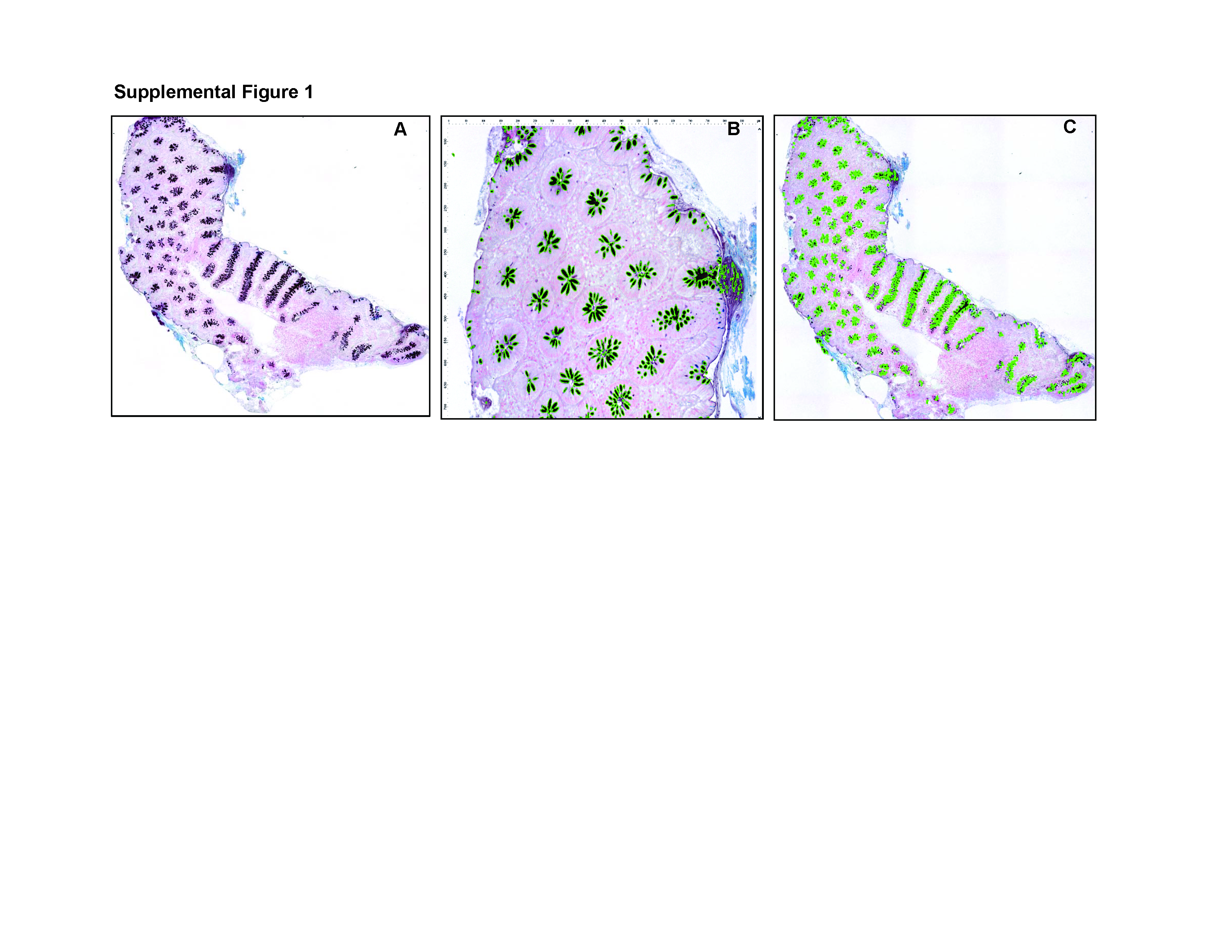

Supplement: Figure S1 — Image analysis of mucin area. A) Images were captured at 200× magnification using a Zeiss Axiovert 200 M Microscope and Axiovision 4.5 software. The MosaiX module was used to scan the entire area of the section at 200× magnification and generate a single image. B) The Automeasure module was used to select and quantify an area of sulfomucin (outlined in green) based on parameters that were used to define sulfomucin staining. C) Selection by software (in green) of total sulfomucin area in the entire section. (TIF) [file pone.0024447.s001.tif]
